# miR-16-5p aggravates sepsis-associated acute kidney injury by inducing apoptosis

**DOI:** 10.1080/0886022X.2024.2322688

**Published:** 2024-03-06

**Authors:** Han Li, Junyan Duan, Tongtong Zhang, Yingjie Fu, Yue Xu, Hongjun Miao, Xuhua Ge

**Affiliations:** a Department of Emergency/Critical Medicine, Children’s Hospital of Nanjing Medical University, Nanjing, PR China; b Jiangsu Key Laboratory of Children’s Major Disease Research, Jiangsu, PR China; c Department of Pediatrics, Changzhou Second Peoples Hospital Affiliated to Nanjing Medical University, Changzhou, PR China

**Keywords:** microRNAs, miR-16-5p, apoptosis, sepsis-associated acute kidney injury, potential biomarker

## Abstract

Sepsis-associated acute kidney injury (S-AKI) is a common disease in pediatric intensive care units (ICU) with high morbidity and mortality. The newly discovered results indicate that microRNAs (miRNAs) play an important role in the diagnosis and treatment of S-AKI and can be used as markers for early diagnosis. In this study, the expression level of miR-16-5p was found to be significantly upregulated about 20-fold in S-AKI patients, and it also increased by 1.9 times in the renal tissue of S-AKI mice. Receiver operating characteristic (ROC) curve analysis showed that miR-16-5p had the highest predictive accuracy in the diagnosis of S-AKI (AUC = 0.9188). *In vitro*, the expression level of miR-16-5p in HK-2 cells treated with 10 μg/mL lipopolysaccharide (LPS) increased by more than 2 times. In addition, LPS-exposed renal tissue and HK-2 cells lead to upregulation of inflammatory cytokines IL-6, IL-1β, TNF-a, and kidney damage molecules kidney injury molecule-1 (KIM-1), neutrophil gelatinase-associated lipocalin (NGAL). However, inhibition of miR-16-5p significantly mitigated LPS expose-mediated kidney injury and inflammation. Furthermore, LPS-exposed HK-2 cells increased more than 1.7-fold the expression levels of Bax and caspase-3, decreased 3.2-fold the expression level of B-cell lymphoma-2 (Bcl-2), and significantly promoted the occurrence of apoptosis. MiR-16-5p mimic further increased LPS-induced apoptosis in HK-2 cells. Nevertheless, inhibition of miR-16-5p significantly attenuated this effect. In summary, up-regulation of miR-16-5p expression can significantly aggravate renal injury and apoptosis in S-AKI, which also proves that miR-16-5p can be used as a potential biomarker to promote early identification of S-AKI.

## 1. Introduction

Sepsis is a multiorgan dysfunction caused by an unbalanced host response to infection [1], with acute kidney injury (AKI) being a common complication in patients with sepsis in the intensive care unit (ICU) [2]. A European multicenter cohort study found that sepsis-associated AKI (S-AKI) in the ICU had a 54% incidence and was associated with a higher mortality rate [3]. Prevention of S-AKI is difficult, and most patients usually seek treatment only when they have obvious clinical symptoms. Thus, early identification helps to provide supportive treatment and limit disease progression for AKI patients [4]. The current diagnosis of AKI is largely dependent on traditional markers such as serum creatinine (Scr) and blood urea nitrogen (BUN). However, the sensitivity and accuracy of these markers remain to be verified, and it is difficult to identify AKI early [5]. Therefore, it is urgent to seek specific diagnostic markers for the early diagnosis and clinical treatment of S-AKI.

MicroRNAs (miRNAs) are small, highly conserved noncoding RNAs that regulate gene expression in animals and plants [6,7]. It mainly inhibits messenger RNA (mRNA) through targeted binding to the 3′ untranslated region (UTR), thereby reducing the expression of target genes [8,9]. Multiple studies have shown that in S-AKI, a variety of miRNAs have anti-inflammatory or anti-apoptotic activities and participate in pathophysiological processes of the disease by activating signaling molecules [10]. Liu et al. [11] found that miR-452 induces the occurrence of S-AKI through activation of nuclear factor kappa beta (NF-κB). Other studies have shown that miR-19b-3p secreted by renal tubular epithelial cells induces renal tubular inflammation by targeting the NF-κB/suppressor of cytokine signaling (SOCS)-1 axis to mediate macrophage activation [12]. Among them, the role of miR-16-5p in a variety of diseases is being recognized. MiR-16-5p can promote apoptosis and autophagy of cardiomyocytes by targeting PTPN4 [13]. In non-small cell carcinoma, miR-16-5p induces cancer cell autophagy and apoptosis by inhibiting BMI1 or mTOR [14]. In addition, miR-16-5p plays a significant role in the kidney and is involved in the occurrence and development of a variety of kidney diseases. In autosomal dominant polycystic kidney disease (ADPKD), miR-16-5p showed significant differences and was selected as the best circulating biomarker for ADPKD [15]. Duan et al. [16] confirmed that miR-16-5p can inhibit podocyte apoptosis and protect diabetic nephropathy caused by hyperglycemia by down-regulating the expression of vascular endothelial growth factor A. In addition, in S-AKI, miR-16-5P induces WNK4 ubiquitination by regulating CUL3, thus disrupting the balance of renal reabsorption and secretion [17,18]. It has also been found that miR-16-5p mediates macrophage activation to promote the progression of SI-AKI [19]. Hence, it can be seen that miR-16-5p plays an important role in the pathophysiological process of S-AKI.

Currently, the specific mechanism of miR-16-5p in AKI remains unclear. In periodontitis, tumor necrosis factor-α (TNF-α) upregulates the expression of miR-16-5p so that miR-16-5p induces apoptosis of human gingival epithelial cells through the BTB domain and CNC homolog 2 [20]. Inhibition of miR-16-5p expression can induce the protective pathway of activating transcription factor 6 in cells, thereby alleviating apoptosis and protecting the heart from oxidative stress-mediated damage [21]. Therefore, miR-16-5p can significantly promote cell apoptosis.

At present, there are few domestic and foreign studies on the role of miR-16-5p in S-AKI. In this study, we found that miR-16-5p mediated apoptosis in S-AKI. In addition, miR-16-5p can be used as a potential biomarker to contribute to the early identification of diseases and provide a new research direction for the clinical treatment of S-AKI.

## 2. Materials and methods

### 2.1. Clinical samples

From June 2020 to December 2022, 18 children with healthy physical examinations and 19 children diagnosed with S-AKI in the PICU were selected from the Children’s Hospital of Nanjing Medical University. Peripheral blood of healthy children and S-AKI children was collected and frozen at −80 °C for subsequent RNA extraction and real-time fluorescence quantitative PCR (qRT–PCR). At the same time, blood biochemistry and other tests were performed on the children. The study was conducted in accordance with the principles of the Declaration of Helsinki and approved by the Ethics Committee of the Children’s Hospital of Nanjing Medical University (approval number: 202008056-1), and informed consent was obtained from all parents of the children.

### 2.2. Animals

This animal study was approved by the Experimental Animal Ethics Committee of Nanjing Medical University (approval number: 20210229), all methods were carried out in accordance with relevant guidelines and regulations. This study was carried out in compliance with the ARRIVE guidelines. The male C57BL/6 mice required for this experiment were purchased and raised in the Animal Experimental Center of Nanjing Medical University. During the experiment, all mice were allowed to eat and drink freely with a 12:12-h light-dark cycle.

Eight-week-old mice were randomly divided into two groups: the control group and the lipopolysaccharide (LPS) group. Mice in the LPS group were given a single intraperitoneal injection of LPS (50 mg/kg, L2880-100, Sigma-Aldrich, St. Louis, MO), and mice in the control group were given an equal dose of normal saline. Twelve hours after modeling, the mice were euthanized, and serum and kidney tissue were retained. Mouse kidney tissue was placed in a 4% paraformaldehyde or −80 °C freezer for follow-up experiments.

### 2.3. Scr and BUN determination

Scr levels were calculated by measuring the absorbance of the serum sample with a microplate reader according to the manufacturer’s instructions for the Scr kit (C011-2-1, Jiancheng, China). BUN levels were determined according to the manufacturer’s instructions for the BUN assay kit (11668019, Jiancheng, China).

### 2.4. Hematoxylin-eosin (HE) staining and tubular injury scoring

Mouse kidney tissue was fixed with 4% paraformaldehyde and embedded in paraffin wax. The embedding block was sliced 3 μm. The slides were stained with hematoxylin for 5 min and eosin for 2 min and sealed with neutral gum at room temperature. Five visual fields were randomly selected from the mouse kidney sections for optical microscope observation, and the images were collected for analysis. The damaged area of mouse renal tubules was calculated according to the renal tubule injury score, including 0 points: normal shape; 1 point: damaged area ≤25%; 2 points: damaged area >25–50%; 3 points: damaged area >50–75%; and 4 points: damaged area >75–100%.

### 2.5. Immunohistochemistry (IHC)

Mouse kidney tissues were fixed in 4% paraformaldehyde and then embedded and sectioned at 4 μm. The mouse kidney tissues were stained according to the manufacturer’s instructions of the IHC kit (CK0062, Signalway Antibody LLC, Greenbelt, MD). The antibodies used mainly included kidney injury molecule-1 (KIM-1) (10 μg/mL, NBP1-76701, Novus, St. Charles, MO), B-cell lymphoma-2 (Bcl-2) (1:500, ab182858, Abcam, Cambridge, MA), BCL2-associated X protein (Bax) (1:1000, 50599-2-lg, ProteinTech, Wuhan, China), and cleaved caspase-3 (1:2000, 9664S, Cell Signaling Technology, Danvers, MA). Visualization was performed using a light microscope.

### 2.6. Enzyme-linked immunosorbent assay (ELISA)

Serum levels of proinflammatory factors, including interleukin-6 (IL-6) (EK203/3-96, Multi sciences, Hangzhou, China) and interleukin-1β (IL-1β) (EK201B/3-96, Multi sciences, China) were determined by ELISA kits according to the manufacturer’s instructions.

### 2.7. Cell culture

HK-2 cells from the human proximal renal tubular epithelial cell line were obtained from the American Type Culture Collection (ATCC, Rockville, MD). The cells were cultured in Dulbecco’s modified Eagle medium (DMEM)/F12 medium (C11330500BT, Gibco, Carlsbad, CA) containing 10% fetal bovine serum (10099141 C, Gibco, USA), 100 U/mL penicillin and 0.1 mg/mL streptomycin. When the cell density reached 80–90%, the cells were given 5 μg/mL or 10 μg/mL LPS, respectively. After 24 h of LPS stimulation, the cells were collected for subsequent study.

### 2.8. Cell transfection

When the density of cells inoculated in the 6-well plate reached 60–70%, transfection complexes were prepared by dissolving transfection reagent Lipofectamine 2000 (11668019, Thermo Fisher, Waltham, MA) and miR-16-5p mimic, miR-16-5p inhibitor and the corresponding negative control (GenePharma, Shanghai, China) in serum-free DMEM-F12 medium. After incubation at room temperature for 20 min, the transfection complexes were dropped into a six-well plate. The six-well plates were placed in a 37 °C incubator for 4 h. Then, the cell culture medium was changed to complete medium. After 24 h, LPS was administered to treat the cells for 24 h. The cells were collected to detect the transfection efficiency by qRT–PCR and carry out the next experiment.

### 2.9. Cell counting kit-8 (CCK-8) assay

The cells were inoculated in 96-well plates, and cell viability was measured using CCK-8 (K1018-10, APExBIO, Houston, TX). The absorbance of the cells at 450 nm was measured on a microplate reader. The absorbance of the cells in the experimental group was compared with that of the control group.

### 2.10. Flow cytometry analysis

HK2 cells at a density of 1 × 10^5^ cell/mL were collected, and the supernatant was discarded after cell suspension. The experiments were then carried out according to the instructions of the FITC Annexin V Apoptosis Detection kit (556547, BD Biosciences, Franklin Lakes, NJ). Finally, the level of cell apoptosis was detected by flow cytometry using CytExpert software.

### 2.11. qRT–PCR

Total RNA from HK-2 cells and kidney tissues was extracted by TRIzol (9109, Takara, Beijing, China). mRNA was reverse transcribed into cDNA using a reverse transcription kit (R222-01, Vazyme, China). After that, qRT–PCR was performed with qPCR SYBR Green Master Mix (Q131-02, Vazyme, China). Primers for each molecule are shown in Table 1.

**Table 1.** Primer sequences.

Gene	Forward Primer (5′–3′)	Reverse Primer (5′–3′)
Mouse KIM-1	ACATATCGTGGAATCACAACGAC	ACTGCTCTTCTGATAGGTGACA
Mouse NGAL	GCAGGTGGTACGTTGTGGG	CTCTTGTAGCTCATAGATGGTGC
Mouse TNF-α	CAGGCGGTGCCTATGTCTC	CGATCACCCCGAAGTTCAGTAG
Mouse IL-6	GGCAATTCTGATTGTATG	GACTCTGGCTTTGTCTTT
Mouse IL-1β	CAACCAACAAGTGATATTCTCCATG	GATCCACACTCTCCAGCTGCA
Mouse miR-16-5P	CGCGTAGCAGCACGTAAATA	AGTGCAGGGTCCGAGGTATT
Mouse U6	CTCGCTTCGGCAGCACATATACT	ACGCTTCACGAATTTGCGTGTC
Mouse GAPDH	AAGAAGGTGGTGAAGCAGG	GAAGGTGGAAGAGTGGGAGT
Human KIM-1	TGGCAGATTCTGTAGCTGGTT	AGAGAACATGAGCCTCTATTCCA
Human NGAL	GAAGTGTGACTACTGGATCAGGA	ACCACTCGGACGAGGTAACT
Human TNF-α	GAGGCCAAGCCCTGGTATG	CGGGCCGATTGATCTCAGC
Human IL-6	ACTCACCTCTTCAGAACGAATTG	CCATCTTTGGAAGGTTCAGGTTG
Human IL-1β	ATGATGGCTTATTACAGTGGCAA	GTCGGAGATTCGTAGCTGGA
Human Bcl-2	GGTGGGGTCATGTGTGTGG	CGGTTCAGGTACTCAGTCATCC
Human miR-16-5P	CGCGTAGCAGCACGTAAATA	AGTGCAGGGTCCGAGGTATT
Human GAPDH	TGTTGCCATCAATGACCCCTT	CTCCACGACGTACTCAGCG

### 2.12. Western blotting analysis

HK-2 cells and mouse kidney tissue were lysed in RIPA lysis buffer (P0013B, Beyotime, China) containing a protease inhibitor to prepare protein samples. The sample was transferred to a PVDF membrane after electrophoresis by SDS–PAGE. The PVDF membrane was blocked with 5% skim milk for 2 h and then coincubated with primary antibody at 4 °C overnight. The antibodies used in this experiment were anti-KIM-1 antibody (4 μg/mL, NBP1-76701, Novus), anti-neutrophil gelatinase-associated lipocalin (NGAL) antibody (1:500, 53213, Signalway Antibody LLC), anti-Bax antibody (1:1000, 50599-2-lg, ProteinTech, China), anti-Bcl-2 antibody (1:1000, ab182858, Abcam), and anti-cleaved caspase-3 antibody (1:1000, 9664S, Cell Signaling Technology). After incubation with horseradish peroxidase-labeled secondary antibodies, the images were imaged by exposure on a gel imager and analyzed for grayscale values using Image Lab software.

### 2.13. Statistical analysis

GraphPad Prism version 8.0 software (La Jolla, CA) was used to process the data. A t test for independent samples was used for comparisons between two groups, and one-way ANOVA was used for comparisons between multiple groups. *p* < 0.05 indicated that the difference was statistically significant.

## 3. Results

### 3.1. Serum levels of miR-16-5p were increased in S-AKI patients

**Figure 1.**
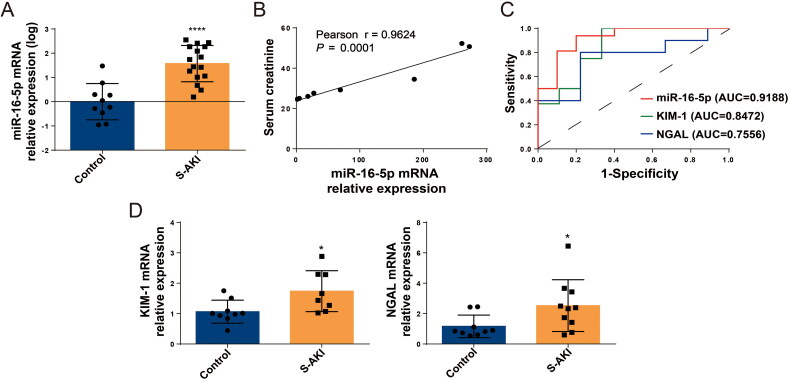
Serum levels of miR-16-5p were increased in S-AKI patients. (A) MiR-16-5p levels in healthy children and children with S-AKI. (B) Analysis of the linear correlation between miR-16-5p and Scr levels in children with S-AKI. (C) The receiver operating characteristic curve of miR-16-5p, KIM-1 and NGAL predicted S-AKI. The AUC were 0.9188, 0.8472, and 0.7556, respectively. (D) qRT–PCR analysis of serum KIM-1 and NGAL levels in healthy children and S-AKI children. *n* ≥ 9, *p < 0.05, **p < 0.01.

We first tested serum samples from healthy children and patients with S-AKI and collected clinical data from the children. In the sera of children with S-AKI, the expression level of miR-16-5p was significantly increased by about 20-fold, and there was a linear positive correlation between miR-16-5p and increased Scr levels (shown in Figure 1(A,B)). The sensitivity and specificity of miR-16-5p, KIM-1, and NGAL in diagnosing S-AKI were evaluated using receiver operating characteristic (ROC) curves and the area under curve (AUC). The AUC was 0.9188, 0.8472, and 0.7556, respectively, which suggested that miR-16-5p had the highest prediction accuracy in the diagnosis of S-AKI (shown in Figure 1(C)). Meanwhile, through qRT–PCR detection, we confirmed that the expression levels of the kidney damage molecules KIM-1 and NGAL were significantly increased in the serum of S-AKI patients (shown in Figure 1(D)). This proved that miR-16-5p was significantly elevated in S-AKI.

### 3.2. Overexpression of miR-16-5p aggravated LPS-induced S-AKI

**Figure 2.**
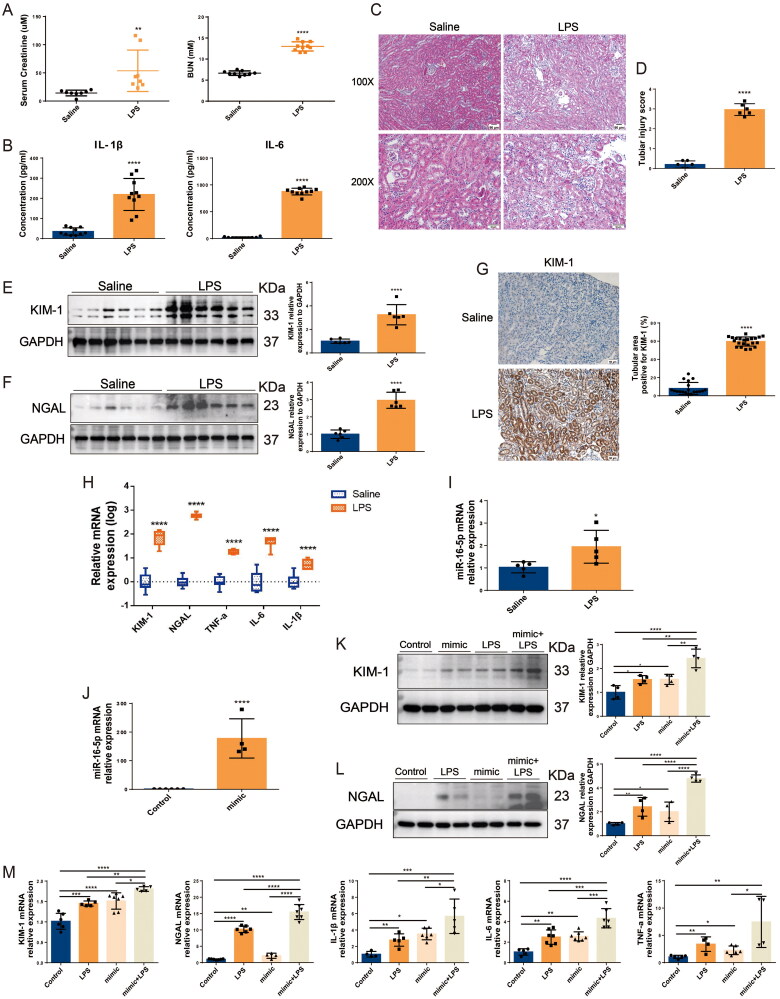
Overexpression of miR-16-5p aggravated LPS-induced S-AKI. (A) Scr and BUN levels after 12 h of treatment with normal saline and LPS (*n* ≥ 8). (B) Serum inflammatory factor levels after saline and LPS treatment for 12 h by ELISA (*n* = 10). (C and D) HE staining images and pathological scores of mouse kidney tissue treated with normal saline and LPS (*n* = 6). Scale bar, 50 μm. (E and F) Expression levels and quantitative analysis of KIM-1 and NGAL in mouse kidney tissues by western blotting (*n* = 6). (G) Expression levels and quantification of KIM-1 in mouse kidney tissues by IHC. Scale bar, 50 μm. (H and I) qRT–PCR analysis of KIM-1, NGAL, inflammatory cytokines and miR-16-5p levels in mouse kidney tissue (*n* ≥ 5). (J) qRT–PCR analysis of miR-16-5p levels in miR-16-5p-overexpressing HK-2 cells (*n* ≥ 4). (K and L) Expression levels and quantitative analysis of KIM-1 and NGAL in LPS-stimulated miR-16-5p-overexpressing HK-2 cells by western blotting (*n* = 4). (M) qRT–PCR analysis of KIM-1, NGAL, inflammatory cytokines, and miR-16-5p levels in LPS-stimulated miR-16-5p-overexpressing HK-2 cells (*n* ≥ 4). *p < 0.05, **p < 0.01, ***p < 0.001, ^****^p < 0.0001.

Based on the findings of the above clinical studies, we conducted experiments *in vitro* and *in vivo* to clarify the role of miR-16-5p in S-AKI. The S-AKI mouse model was constructed by LPS treatment. ELISA results showed that the levels of Scr, BUN, and the inflammatory cytokines IL-6 and IL-1β were significantly increased in mice treated with LPS for 12 h (shown in Figure 2(A,B)). HE staining revealed that LPS induced renal tubule injury in mice, including vacuolation, inflammatory cell infiltration, and tubular formation (shown in Figure 2(C,D)). These results indicated that the S-AKI model of mice induced by LPS was successfully constructed. In terms of gene and protein levels, we found that compared with the normal saline group, in addition to the mRNA and protein expression levels of KIM-1, NGAL, and the mRNA expression levels of inflammatory cytokines TNF-a, IL-6, and IL-1β increased significantly in the LPS group (shown in Figure 2(E–H)). In the kidney tissues of S-AKI mice, the expression level of miR-16-5p was also significantly increased by 1.9 times (shown in Figure 2(I)). To further clarify the role of miR-16-5p, a miR-16-5p mimic was successfully constructed in HK-2 cells. *In vitro* experiments revealed that the miR-16-5p mimic significantly increased the expression levels of inflammatory cytokines and the kidney damage molecules KIM-1 and NGAL in LPS-induced HK-2 cells (shown in Figure 2(J–M)), thereby aggravating LPS-induced HK-2 cell damage. These results suggest that overexpression of miR-16-5p can significantly aggravate LPS-induced S-AKI.

### 3.3. Knockdown of miR-16-5p significantly alleviated S-AKI in HK2 cells induced by LPS

**Figure 3.**
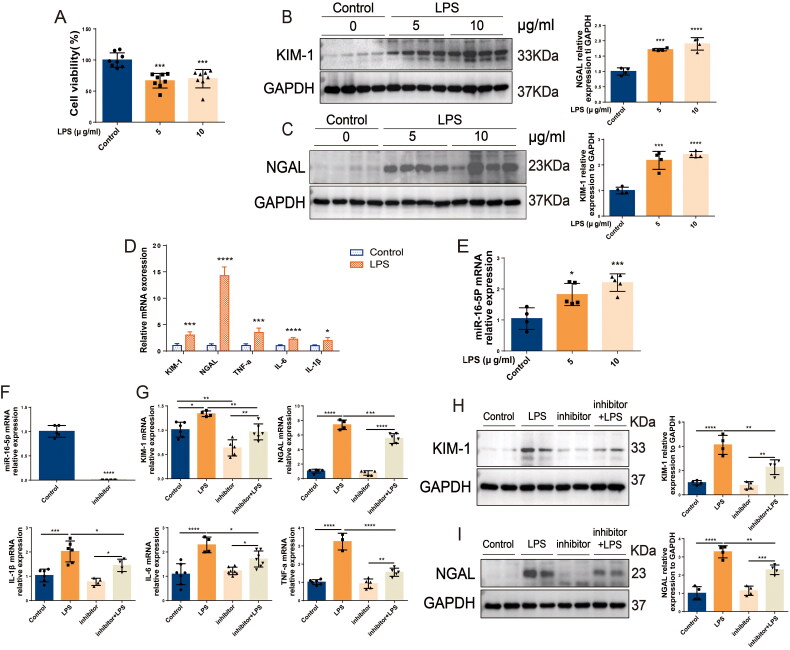
Knockdown of miR-16-5p significantly alleviated S-AKI in HK-2 cells induced by LPS. (A) Cell viability of HK-2 cells treated with LPS (*n* = 8). (B and C) Expression levels and quantitative analysis of KIM-1 and NGAL in HK-2 cells treated with different concentrations of LPS by western blotting (*n* = 4). (D and E) qRT–PCR analysis of KIM-1, NGAL, inflammatory cytokines and miR-16-5p levels in HK-2 cells treated with LPS (*n* ≥ 4). (F) qRT–PCR analysis of miR-16-5p levels in HK-2 cells that inhibit miR-16-5p (*n* ≥ 4). (G) qRT–PCR analysis of KIM-1, NGAL, and inflammatory factor levels in LPS-stimulated miR-16-5p knockdown HK-2 cells (*n* ≥ 4). (H and I) Expression levels and quantitative analysis of KIM-1 and NGAL in LPS-stimulated miR-16-5p knockdown HK-2 cells by western blotting (*n* = 4). *p < 0.05, **p < 0.01, ***p < 0.001, ^****^p < 0.0001.

In HK-2 cells, LPS stimulation caused a significant decrease in cell viability (shown in Figure 3(A)). Western blotting results showed that the expression levels of KIM-1 and NGAL in HK-2 cells in the LPS group were significantly higher than those in the control group. The expression levels of KIM-1 and NGAL were significantly increased with increasing LPS concentration, and the difference was most significant at a concentration of 10 μg/mL LPS (shown in Figure 3(B,C)). In addition, 10 μg/mL LPS treatment not only caused a significant increase in the mRNA levels of IL-6, IL-1β, TNF-a, KIM-1, and NGAL in HK-2 cells but also led to a more than 2-fold significant increase in the expression level of miR-16-5p (shown in Figure 3(D,E)). To further clarify the role of miR-16-5p, a miR-16-5p inhibitor was successfully constructed in HK-2 cells. *In vitro*, inhibition of miR-16-5p significantly decreased the expression of KIM-1 and NGAL as well as the inflammatory cytokines IL-6, IL-1β, and TNF-a in LPS-induced HK-2 cells (shown in Figure 3(F—I)). This suggests that inhibition of miR-16-5p can effectively protect LPS-induced HK-2 cells from S-AKI.

### 3.4. In S-AKI, upregulation of miR-16-5p improves apoptosis of renal tubules

**Figure 4.**
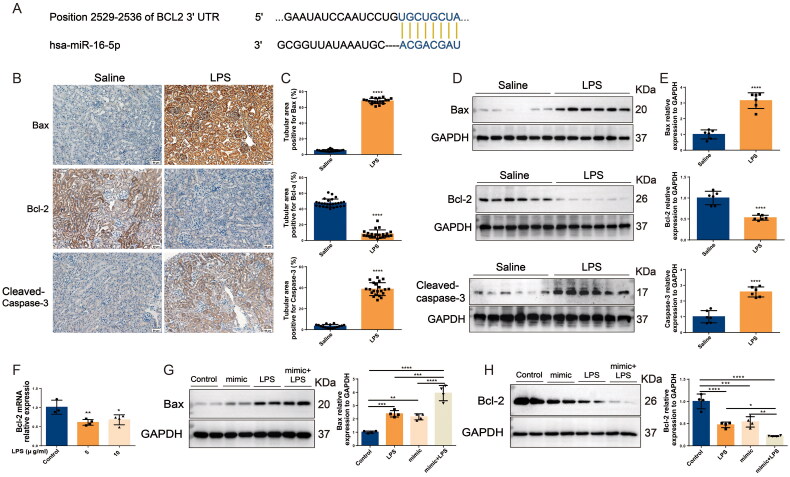
In S-AKI, upregulation of miR-16-5p improves apoptosis of renal tubules. (A) Targeted binding sequence of hsa-miR-16-5p to the 3’-UTR end of bcl-2. (B and C) Expression levels and quantification of bax, bcl-2, and caspase-3 in mouse kidney tissues by IHC. Scale bar, 50 μm. (D and E) Expression levels and quantitative analysis of bax, bcl-2, and caspase-3 in mouse kidney tissues by western blotting (*n* = 6). (F) qRT–PCR analysis of bcl-2 expression levels in HK-2 cells treated with different concentrations of LPS (*n* ≥ 3). (G and H) Expression levels and quantification of bax and bcl-2 in LPS-stimulated miR-16-5p overexpressing HK-2 cells by western blotting (*n* = 4). *p < 0.05, **p < 0.01, ***p < 0.001, ^****^p < 0.0001.

The above studies confirmed that miR-16-5p can significantly promote the occurrence of S-AKI. To further clarify the role of miR-16-5p, we predicted by the TargetScan database that a conserved binding site with miR-16-5p was found at the 3′ UTR end of BCL2 (shown in Figure 4(A)), suggesting that the downstream target of miR-16-5p is BCL2, which is closely related to apoptosis. Subsequently, both IHC and Western blotting results showed that in LPS-induced S-AKI mice, the levels of Bax and caspase-3 were significantly increased, while the levels of Bcl-2 were significantly decreased (shown in Figure 4(B–E)). qRT–PCR results demonstrated that compared with the control group, the level of Bcl-2 in HK-2 cells in the LPS group was downregulated (shown in Figure 4(F)). We further explored the role of miR-16-5p in LPS-induced S-AKI. It was revealed that overexpression of miR-16-5p further promoted the upregulation of Bax levels and downregulation of Bcl-2 levels induced by LPS stimulation (shown in Figure 4(G,H)). This suggests that miR-16-5p can promote the apoptosis of renal tubules in S-AKI.

### 3.5. Inhibition of miR-16-5p alleviates HK-2 cell apoptosis

**Figure 5.**
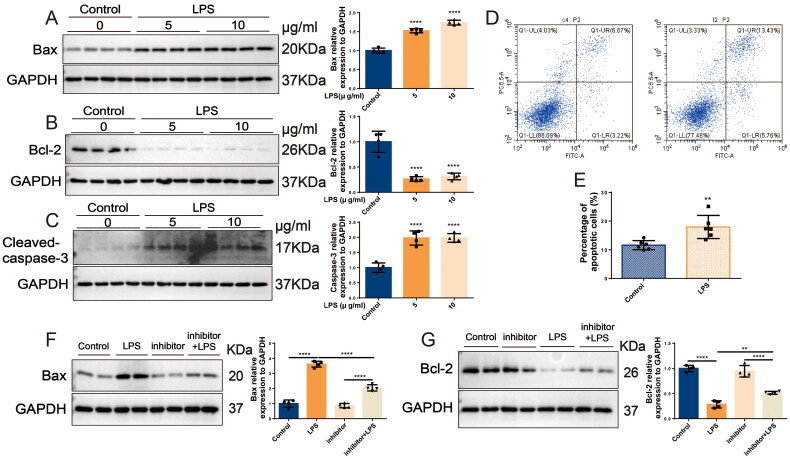
Inhibition of miR-16-5p alleviates HK-2 cell apoptosis. (A-C) Expression levels and quantification of bax, bcl-2, and caspase-3 in HK-2 cells treated with different concentrations of LPS by western blotting (*n* = 4). (D and E) Apoptosis levels and quantification of apoptotic cells in LPS-treated HK-2 cells by flow cytometry (*n* = 6). (F and G) Expression levels and quantification of bax and bcl-2 in LPS-stimulated miR-16-5p knockdown HK-2 cells by western blotting (*n* = 4). **p < 0.01, ^****^p < 0.0001.

In HK-2 cells, LPS was observed to induce a significant increase in intracellular Bax and caspase-3 by more than 1.7-fold and a significant decrease in Bcl-2 by 3.2-fold (shown in Figure 5(A–C)). Flow cytometry detected that HK-2 cell apoptosis was significantly increased in the LPS group compared with the control group (shown in Figure 5(D,E)). In contrast, knockdown of miR-16-5p significantly attenuated the LPS-induced increase in Bax levels and decrease in Bcl-2 levels (shown in Figure 5(F,G)). These results suggest that knockdown of miR-16-5p can significantly reduce LPS-induced apoptosis in HK-2 cells.

## 4. Discussion

S-AKI is a common life-threatening complication and a leading cause of death in ICU patients [22]. In recent years, miRNAs have become increasingly widespread in the study of AKI. A large amount of evidence suggests that in S-AKI, multiple miRNAs are involved in damage repair and apoptosis of the disease by binding to target genes, providing more directions for disease treatment [23]. Sang et al. found that miR-214 activates the protein kinase B/mammalian target of rapamycin signaling pathway by targeting phosphatase and tensin homolog, alleviating oxidative stress and autophagy, thereby improving S-AKI [24]. Ding et al. [25] demonstrated that miR-103a-3p alleviates inflammation and apoptosis of renal tubular epithelial cells by regulating chemokine (c-x-c motif) ligand 12 (CXCL12). MiR-22 suppresses the inflammatory response and attenuates S-AKI by inhibiting the high-mobility group box 1/toll-like receptor 4/NF-κB pathway [26]. In our study, an important role of miR-16-5p in S-AKI was demonstrated. In S-AKI patients, the differentially expressed miRNA we screened was miR-16-5p, and its expression level was significantly increased. In kidney tissues of S-AKI mice and LPS-stimulated HK2 cells, we also verified that miR-16-5p expression was significantly elevated. This suggests that miR-16-5p may serve as a potential biomarker for S-AKI.

In the TargetScan database, we predicted that the traditional binding site of miR-16-5p was BCL-2. A literature review identified that BCL-2 is a major regulatory factor located in mitochondria that can inhibit apoptosis and is closely related to apoptosis [27,28]. Apoptosis, as a form of cell death, has been proven to be closely related to the occurrence and development of many diseases [29]. More and more studies have shown that apoptosis plays an important role in S-AKI. Huang et al. [30] found that apoptosis of renal tubular epithelial cells plays an important role in AKI, and calcium/calmodulin-dependent protein kinase II may induce apoptosis of renal tubular epithelial cells through the yes-associated protein/nuclear factor of activated T cells 2 pathway, thus promoting AKI. LPS can activate cathepsin B by promoting lysosomal membrane permeabilization, leading to mitochondrial apoptosis, which causes the apoptosis of HK-2 cells and aggravates AKI [31]. In addition, miR-16-5p is involved in a variety of pathophysiological processes in the body, such as cellular oxidative stress, inflammatory response, and apoptosis. Among them, it is closely related to apoptosis [32,33]. In Alzheimer’s disease, amyloid beta drives increased expression of miR-16-5p, which induces apoptosis through downregulation of Bcl-2 [34]. In diabetes, a dual luciferase reporter gene assay verified that miR-16-5p and CXCL10 interact and that both are involved in the process of apoptosis [35]. It has also been found that miR-16-5p promotes apoptosis in breast cancer by targeting the actin-binding protein anillin to regulate the cell cycle [36]. However, the role of miR-16-5p in apoptosis in S-AKI remains unclear. In view of the above findings, we designed experiments to investigate the role of miR-16-5p in apoptosis in S-AKI.

**Figure 6.**
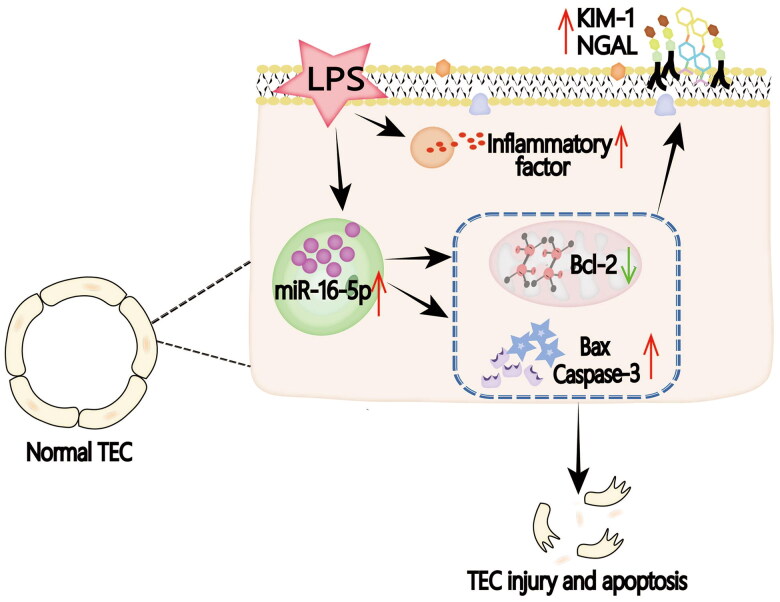
Graphical abstract. In the LPS-induced S-AKI model, the expression of miR-16-5p is significantly upregulated by mediating the occurrence of apoptosis, thus aggravating S-AKI.

In our study, we used LPS intervention to construct the classical model of S-AKI. In this study, we found that in LPS-treated mice and HK-2 cells, not only the expression of inflammatory factors IL-6, TNF-α, IL-1β, and renal injury molecules KIM-1 and NGAL but also the expression of apoptosis-related indicators Bax and caspase3 were significantly increased. Bax, caspase3, and Bcl-2 are commonly used markers to reflect the level of apoptosis [37]. When cells undergo apoptosis, the levels of Bax and caspase3 increase, while the level of Bcl-2 decreases [38]. At the same time, we also found that the overexpression of miR-16-5p increased the protein levels of the kidney damage molecules KIM-1 and NGAL and the apoptosis-related indicator Bax protein, but decreased the level of Bcl-2, suggesting that the increase in miR-16-5p aggravated the cell damage and apoptosis of HK-2 cells. However, inhibition of miR-16-5p attenuated the elevations of KIM-1, NGAL, and Bax induced by LPS stimulation, suggesting that inhibition of miR-16-5p could block LPS-induced kidney injury and apoptosis (shown in Figure 6).

MiRNAs are increasingly being studied in diseases. We verified that miR-16-5p is significantly upregulated during S-AKI at multiple levels. This suggests that miR-16-5p can be used as a potential biomarker, providing a new possibility for the monotonous diagnostic method of S-AKI, which is a promising diagnostic scheme. However, the limitation of this study is that it only verified the important role of miR-16-5p in S-AKI cell apoptosis without clarifying the target of miR-16-5p and its specific mechanism of regulating cell apoptosis. In the future, we will conduct dual luciferase experiments to clarify the target of miR-16-5p in cell apoptosis and conduct further experiments to explore its specific mechanism.

## 5. Conclusion

In summary, in *in vitro* and *in vivo* studies, we confirmed that miR-16-5p plays a key role in S-AKI. Inhibition of miR-16-5p expression could effectively ameliorate kidney injury, and its possible mechanism of action is closely related to the promotion of apoptosis by miR-16-5p. Our study suggests that miR-16-5p could be used as a potential biomarker and therapeutic target for S-AKI.

## Data Availability

The original contributions presented in the study are included in the article, and further inquiries can be directed to the corresponding authors.
